# Supplementary material for: Trends in Online Search Activity and the Correlation with Daily New Cases of Monkeypox among 102 Countries or Territories
Source: Int J Environ Res Public Health. 2023 Feb 15;20(4):3395. doi: 10.3390/ijerph20043395 (PMC9963132; doi:10.3390/ijerph20043395)
Supplement: Supplementary file 1 [file ijerph-20-03395-s001.zip › ijerph-2139281-supplementary.pdf]

Supplementary materials

This file formed part of the original submission.

Table S1 The value of β1, β2 and β1+β3 among 194 countries or territories............1

Figure S1 Time-lag correlations of global online search activity and daily new cases 6

Table S2 Time-lag correlations of online search activity and daily new cases in 102 countries or territories 7

Table S1. The value of β_1, β_2 and $\beta_1+\beta_3$ among 194 countries or territories.

Country or territories	Before		Change		After			
	β1 value	P	β2 value	P	β3 value	P	β1+β3 value	Region
World	-0.164	0.062	42.845	<0.001	-0.631	<0.001	-0.795	
Afghanistan	0.119	0.204	-0.928	0.885	-0.248	0.073	-0.128	Asia
Albania	0.000	0.997	5.293	0.240	-0.171	0.077	-0.171	Europe
Algeria	-0.134	0.065	7.376	0.137	0.044	0.678	-0.090	Africa
American Samoa	0.133	0.188	3.889	0.576	-0.316	0.035	-0.182	Oceania
Andorra	-0.136	0.067	8.158	0.119	0.176	0.113	0.041	Europe
Angola	-0.105	0.267	9.942	0.127	-0.012	0.930	-0.118	Africa
Antigua & Barbuda	0.094	0.294	8.194	0.181	-0.294	0.026	-0.200	North America
Argentina	-0.142	0.035	11.551	0.013	-0.061	0.537	-0.203	South America
Armenia	-0.032	0.606	3.134	0.455	0.031	0.729	0.000	Asia
Aruba	0.037	0.679	0.669	0.914	-0.150	0.261	-0.112	North America
Australia	-0.107	0.016	9.716	0.001	-0.052	0.427	-0.158	Oceania
Austria	-0.163	0.033	18.896	<0.001	-0.119	0.305	-0.282	Europe
Azerbaijan	0.013	0.872	-0.647	0.907	0.008	0.948	0.021	Asia
Bahamas	0.046	0.693	4.743	0.552	-0.227	0.185	-0.181	North America
Bahrain	-0.089	0.322	12.120	0.051	-0.057	0.667	-0.147	Asia
Bangladesh	-0.176	0.004	6.289	0.132	0.051	0.571	-0.125	Asia
Barbados	0.171	0.030	-12.054	0.025	-0.192	0.094	-0.021	North America
Belarus	-0.050	0.408	7.677	0.065	-0.065	0.461	-0.115	Europe
Belgium	-0.174	0.024	18.813	<0.001	-0.164	0.151	-0.337	Europe
Belize	-0.071	0.309	11.247	0.019	-0.083	0.414	-0.154	North America
Benin	-0.022	0.739	-0.230	0.959	-0.035	0.717	-0.057	Africa
Bermuda	0.192	0.025	-5.270	0.372	-0.298	0.019	-0.106	North America
Bhutan	-0.066	0.487	8.677	0.186	-0.126	0.369	-0.192	Asia
Bolivia	0.162	0.073	-7.573	0.222	-0.194	0.157	-0.032	South America
Bosnia and Herzegovina	0.034	0.729	2.849	0.676	-0.100	0.495	-0.065	Europe
Botswana	0.038	0.561	-3.786	0.397	-0.099	0.303	-0.061	Africa
Brazil	0.127	0.024	50.611	<0.001	-0.917	<0.001	-0.790	South America
Brunei	-0.184	0.081	5.866	0.414	0.189	0.220	0.006	Asia
Bulgaria	-0.163	0.103	12.473	0.069	-0.093	0.527	-0.256	Europe
Burkina Faso	-0.113	0.266	2.993	0.667	0.171	0.252	0.058	Africa
Burundi	0.021	0.691	0.316	0.931	-0.013	0.871	0.009	Africa
Cambodia	-0.141	0.064	25.391	<0.001	-0.251	0.025	-0.392	Asia
Cameroon	-0.060	0.559	0.208	0.976	0.120	0.424	0.060	Africa
Canada	-0.166	0.011	14.849	0.001	-0.138	0.162	-0.304	North America
Cape Verde	0.009	0.883	-2.961	0.458	0.024	0.782	0.032	Africa
Chile	-0.015	0.873	13.244	0.046	-0.206	0.149	-0.221	South America
China	-0.060	0.539	4.415	0.511	0.019	0.894	-0.041	Asia
Colombia	0.019	0.837	31.487	<0.001	-0.628	<0.001	-0.609	South America
Congo	0.114	0.175	-8.036	0.161	-0.101	0.412	0.013	Africa
Costa Rica	-0.228	0.015	22.076	0.001	-0.072	0.597	-0.300	North America
cote d'ivoire	-0.115	0.182	4.992	0.397	0.061	0.630	-0.054	Africa
Croatia	-0.156	0.016	12.509	0.005	-0.052	0.582	-0.209	Europe

Cuba	0.088	0.348	1.092	0.865	-0.253	0.070	-0.165	North America
Curacao	-0.138	0.136	6.788	0.286	0.051	0.707	-0.087	North America
Cyprus	-0.237	0.003	11.194	0.040	0.133	0.247	-0.104	Europe
Czechia	-0.165	0.060	10.970	0.071	0.007	0.954	-0.157	Europe
Democratic Republic of Congo	-0.077	0.239	7.858	0.080	0.032	0.738	-0.046	Africa
Denmark	-0.230	0.002	12.958	0.010	0.045	0.689	-0.185	Europe
Djibouti	0.005	0.943	0.374	0.934	0.013	0.890	0.018	Africa
Dominica	0.039	0.507	-3.934	0.329	-0.025	0.767	0.013	North America
Dominican Republic	-0.027	0.806	16.182	0.032	-0.370	0.023	-0.396	North America
Ecuador	-0.143	0.149	10.901	0.110	0.017	0.906	-0.126	South America
Egypt	-0.162	0.016	12.979	0.005	-0.019	0.844	-0.181	Africa
El Salvador	-0.033	0.751	-0.552	0.939	0.094	0.553	0.061	North America
Equatorial Guinea	-0.077	0.380	3.217	0.593	0.131	0.310	0.054	Africa
Estonia	-0.052	0.557	1.260	0.833	0.023	0.862	-0.029	Europe
eSwatini	0.154	0.029	-14.275	0.003	-0.104	0.311	0.049	Africa
Ethiopia	-0.166	0.086	17.032	0.011	-0.051	0.720	-0.217	Africa
Fiji	-0.007	0.931	0.877	0.875	0.026	0.826	0.019	Oceania
Finland	-0.238	<0.001	15.749	0.001	0.020	0.838	-0.218	Europe
France	-0.058	0.371	15.691	<0.001	-0.344	<0.001	-0.402	Europe
Gabon	0.056	0.480	-9.154	0.091	0.001	0.994	0.056	Africa
Georgia	-0.048	0.464	7.274	0.106	-0.093	0.332	-0.141	Asia
Germany	-0.137	0.102	14.991	0.003	-0.200	0.114	-0.337	Europe
Ghana	-0.080	0.259	-0.057	0.991	-0.054	0.601	-0.134	Africa
Gibraltar	-0.013	0.883	6.023	0.321	-0.067	0.603	-0.081	Europe
Greece	-0.206	0.001	18.669	<0.001	-0.103	0.270	-0.309	Europe
Greenland	-0.013	0.861	1.444	0.786	-0.047	0.681	-0.060	North America
Grenada	-0.005	0.951	1.141	0.850	-0.099	0.444	-0.104	North America
Guadeloupe	-0.044	0.547	-3.817	0.453	0.290	0.008	0.246	North America
Guam	-0.008	0.855	9.590	0.002	-0.139	0.033	-0.147	Oceania
Guatemala	-0.129	0.063	11.403	0.018	0.074	0.485	-0.055	North America
Guinea	0.088	0.198	-8.916	0.057	-0.059	0.555	0.029	Africa
Guinea-Bissau	-0.097	0.200	6.744	0.191	-0.026	0.814	-0.123	Africa
Guyana	-0.017	0.728	4.058	0.236	-0.012	0.873	-0.029	South America
Haiti	-0.028	0.736	13.362	0.018	-0.134	0.266	-0.162	North America
Honduras	-0.092	0.384	4.466	0.541	0.026	0.869	-0.067	North America
Hungary	-0.122	0.163	12.040	0.041	-0.104	0.423	-0.226	Europe
Iceland	0.026	0.790	7.085	0.286	-0.169	0.231	-0.144	Europe
India	-0.038	0.438	18.183	<0.001	-0.334	<0.001	-0.372	Asia
Indonesia	-0.096	0.173	40.298	<0.001	-0.503	<0.001	-0.599	Asia
Iran	-0.168	0.021	9.495	0.057	0.061	0.563	-0.107	Asia
Iraq	-0.131	0.053	2.761	0.550	0.079	0.424	-0.052	Asia
Ireland	-0.274	<0.001	12.744	0.006	0.086	0.385	-0.188	Europe
Israel	-0.185	0.009	15.980	0.001	-0.011	0.918	-0.196	Asia
Italy	-0.149	0.065	32.083	<0.001	-0.443	<0.001	-0.591	Europe
Jamaica	0.051	0.463	1.413	0.769	-0.257	0.013	-0.205	North America
Japan	-0.092	0.172	24.785	<0.001	-0.424	<0.001	-0.516	Asia

Jordan	-0.212	0.005	7.544	0.141	0.125	0.254	-0.086	Asia
Kazakhstan	-0.004	0.964	0.067	0.990	0.081	0.474	0.078	Asia
Kenya	-0.259	<0.001	9.288	0.058	0.107	0.306	-0.152	Africa
Kuwait	-0.139	0.133	13.901	0.029	-0.123	0.365	-0.262	Asia
Kyrgyzstan	0.082	0.301	-7.020	0.197	-0.059	0.611	0.023	Asia
Laos	-0.058	0.584	8.459	0.242	0.024	0.878	-0.034	Asia
Latvia	0.114	0.166	-10.395	0.066	-0.166	0.168	-0.052	Europe
Lebanon	-0.164	0.006	4.678	0.242	0.092	0.281	-0.072	Asia
Lesotho	0.019	0.800	5.931	0.252	-0.177	0.111	-0.158	Africa
Liberia	-0.050	0.656	9.925	0.202	-0.104	0.531	-0.153	Africa
Libya	0.039	0.674	-4.225	0.505	-0.022	0.871	0.017	Africa
Lithuania	-0.107	0.200	10.865	0.063	-0.180	0.146	-0.287	Europe
Luxembourg	0.108	0.082	-5.085	0.233	-0.112	0.240	-0.005	Europe
Madagascar	-0.127	0.006	3.215	0.305	0.123	0.069	-0.004	Africa
Malawi	0.050	0.643	-6.272	0.400	0.013	0.933	0.064	Africa
Malaysia	-0.183	0.017	15.298	0.004	-0.166	0.139	-0.348	Asia
Maldives	-0.004	0.968	3.211	0.604	-0.139	0.294	-0.143	Asia
Mali	0.018	0.862	2.774	0.692	-0.018	0.907	<0.001	Africa
Malta	-0.089	0.102	2.152	0.560	0.090	0.262	0.001	Europe
Martinique	-0.009	0.926	3.915	0.533	-0.089	0.508	-0.097	North America
Mauritania	0.027	0.618	-7.119	0.054	0.130	0.098	0.157	Africa
Mauritius	-0.243	0.020	11.259	0.113	0.089	0.559	-0.154	Africa
Mexico	-0.157	0.017	38.690	<0.001	-0.485	<0.001	-0.642	North America
Moldova	-0.068	0.391	4.281	0.436	0.058	0.620	-0.010	Europe
Mongolia	-0.033	0.689	6.501	0.254	-0.049	0.687	-0.082	Asia
Montenegro	-0.033	0.724	2.356	0.716	-0.023	0.867	-0.056	Europe
Morocco	-0.217	0.001	9.474	0.027	0.112	0.220	-0.105	Africa
Mozambique	0.011	0.896	1.811	0.744	-0.127	0.285	-0.117	Africa
Myanmar	-0.166	0.067	12.513	0.044	-0.073	0.579	-0.239	Asia
Namibia	0.059	0.510	0.218	0.972	-0.195	0.140	-0.136	Africa
Nepal	-0.097	0.184	11.645	0.021	-0.183	0.089	-0.280	Asia
Netherlands	-0.124	0.047	12.158	0.003	-0.125	0.180	-0.249	Europe
New Caledonia	-0.055	0.513	0.625	0.913	0.046	0.706	-0.009	Oceania
New Zealand	-0.026	0.684	2.432	0.583	-0.136	0.152	-0.162	Oceania
Nicaragua	0.003	0.975	-0.434	0.940	-0.010	0.934	-0.008	North America
Niger	0.094	0.228	-3.741	0.483	-0.059	0.605	0.035	Africa
Nigeria	-0.168	0.014	17.203	<0.001	-0.069	0.490	-0.236	Africa
North Macedonia	-0.237	0.004	8.017	0.151	0.174	0.147	-0.064	Europe
Northern Mariana Islands	-0.020	0.824	4.682	0.458	-0.147	0.278	-0.167	Oceania
Norway	-0.172	0.028	15.826	0.002	-0.158	0.171	-0.330	Europe
Oman	-0.076	0.269	6.956	0.138	-0.122	0.226	-0.197	Asia
Pakistan	-0.139	0.009	3.911	0.283	0.077	0.321	-0.062	Asia
Palau	0.231	0.026	-8.925	0.206	-0.282	0.063	-0.051	Oceania
Palestine	-0.072	0.386	-8.042	0.160	0.207	0.093	0.134	Asia
Panama	-0.130	0.141	10.635	0.081	-0.022	0.863	-0.153	North America
Papua New Guinea	0.174	0.084	-6.581	0.338	-0.217	0.140	-0.044	Oceania
Paraguay	0.150	0.096	2.105	0.733	-0.323	0.016	-0.173	South America
Peru	-0.175	0.042	18.814	0.002	-0.059	0.637	-0.234	South America
Philippines	-0.035	0.363	13.820	<0.001	-0.195	0.001	-0.231	Asia
Poland	-0.217	0.004	19.757	<0.001	-0.125	0.257	-0.342	Europe
Portugal	-0.186	0.004	13.750	0.002	-0.104	0.272	-0.289	Europe
Puerto Rico	0.008	0.925	28.610	<0.001	-0.561	<0.001	-0.553	North America
Qatar	-0.231	0.006	20.982	<0.001	-0.117	0.341	-0.348	Asia

Romania	-0.151	0.015	12.087	0.004	-0.073	0.425	-0.224	Europe
Russia	-0.070	0.491	11.633	0.096	-0.149	0.317	-0.219	Europe
Rwanda	0.003	0.976	-2.414	0.687	-0.060	0.642	-0.057	Africa
Saint Kitts and Nevis	0.016	0.872	-1.132	0.870	0.030	0.837	0.047	North America
Saint Lucia	-0.061	0.352	9.820	0.029	-0.140	0.147	-0.200	North America
Saint Vincent and the Grenadines	-0.022	0.676	2.389	0.503	-0.036	0.632	-0.058	North America
Samoa	-0.010	0.911	8.954	0.133	-0.167	0.190	-0.177	Oceania
Saudi Arabia	-0.165	0.033	12.450	0.019	-0.077	0.506	-0.242	Asia
Senegal	0.049	0.433	-2.137	0.618	-0.066	0.472	-0.017	Africa
Serbia	-0.261	0.006	14.900	0.021	0.095	0.490	-0.166	Europe
Seychelles	0.156	0.047	-4.505	0.401	-0.139	0.227	0.017	Africa
Sierra Leone	-0.022	0.795	-0.347	0.951	0.109	0.371	0.088	Africa
Singapore	-0.108	0.247	15.447	0.016	-0.377	0.008	-0.484	Asia
Slovakia	-0.179	0.117	13.147	0.096	-0.054	0.750	-0.234	Europe
Slovenia	-0.112	0.108	4.881	0.290	0.061	0.558	-0.051	Europe
Solomon Islands	0.028	0.743	2.150	0.709	-0.046	0.711	-0.018	Oceania
Somalia	-0.041	0.558	3.713	0.439	-0.081	0.433	-0.122	Africa
South Africa	0.003	0.939	-2.827	0.316	-0.039	0.522	-0.036	Africa
South Korea	0.026	0.734	6.833	0.188	-0.371	0.001	-0.345	Asia
South Sudan	0.056	0.524	-6.235	0.305	-0.088	0.501	-0.031	Africa
Spain	-0.215	0.007	22.086	<0.001	-0.204	0.091	-0.419	Europe
Sri Lanka	-0.048	0.367	20.637	<0.001	-0.323	<0.001	-0.371	Asia
Sudan	-0.183	0.039	1.890	0.754	0.142	0.274	-0.040	Africa
Suriname	0.032	0.584	-4.129	0.305	0.039	0.654	0.071	South America
Sweden	-0.223	0.002	16.490	0.001	-0.038	0.718	-0.261	Europe
Switzerland	-0.037	0.655	11.618	0.034	-0.307	0.018	-0.345	Europe
Syria	-0.027	0.660	2.030	0.624	-0.029	0.744	-0.056	Asia
Taiwan (provinces of China)	-0.057	0.460	6.849	0.196	-0.173	0.127	-0.230	Asia
Tajikistan	0.048	0.445	-4.278	0.326	-0.044	0.639	0.005	Asia
Tanzania	-0.094	0.295	5.840	0.343	-0.016	0.903	-0.110	Africa
Thailand	-0.113	0.241	28.867	<0.001	-0.518	<0.001	-0.631	Asia
The Gambia	-0.074	0.469	9.404	0.180	-0.025	0.867	-0.099	Africa
Timor-Leste	0.040	0.550	-2.247	0.626	-0.048	0.630	-0.007	Asia
Togo	-0.018	0.830	-3.023	0.591	0.069	0.569	0.051	Africa
Tonga	0.022	0.734	3.432	0.440	-0.118	0.215	-0.096	Oceania
Trinidad and Tobago	-0.041	0.574	11.198	0.026	-0.287	0.008	-0.328	North America
Tunisia	-0.151	0.088	5.631	0.353	0.068	0.600	-0.083	Africa
Turkey	-0.149	0.012	10.644	0.009	-0.057	0.509	-0.206	Asia
Turkmenistan	-0.127	0.134	1.653	0.776	0.196	0.118	0.068	Europe
Uganda	0.029	0.747	1.417	0.821	-0.355	0.009	-0.326	Africa
Ukraine	-0.100	0.219	6.460	0.249	-0.047	0.700	-0.147	Europe
United Arab Emirates	-0.210	0.002	7.165	0.121	0.101	0.306	-0.108	Asia
United Kingdom	-0.239	<0.001	11.144	0.008	0.043	0.644	-0.196	Europe
United States	-0.051	0.431	45.423	<0.001	-0.784	<0.001	-0.834	North America
Uruguay	-0.122	0.233	-1.839	0.793	0.179	0.236	0.057	South America
Uzbekistan	-0.021	0.791	11.908	0.032	-0.105	0.377	-0.126	Asia
Vanuatu	-0.041	0.469	0.906	0.816	0.040	0.633	-0.001	Oceania
Venezuela	-0.095	0.276	8.612	0.152	<0.001	0.999	-0.095	South America
Vietnam	-0.052	0.532	22.790	<0.001	-0.440	<0.001	-0.492	Asia
Virgin Islands	-0.038	0.658	-3.860	0.516	0.142	0.264	0.104	North America
Yemen	0.017	0.823	6.583	0.212	-0.233	0.040	-0.216	Asia

Zambia	-0.066	0.283	0.618	0.883	-0.029	0.745	-0.095	Africa
Zimbabwe	0.084	0.336	1.129	0.850	-0.319	0.013	-0.235	Africa

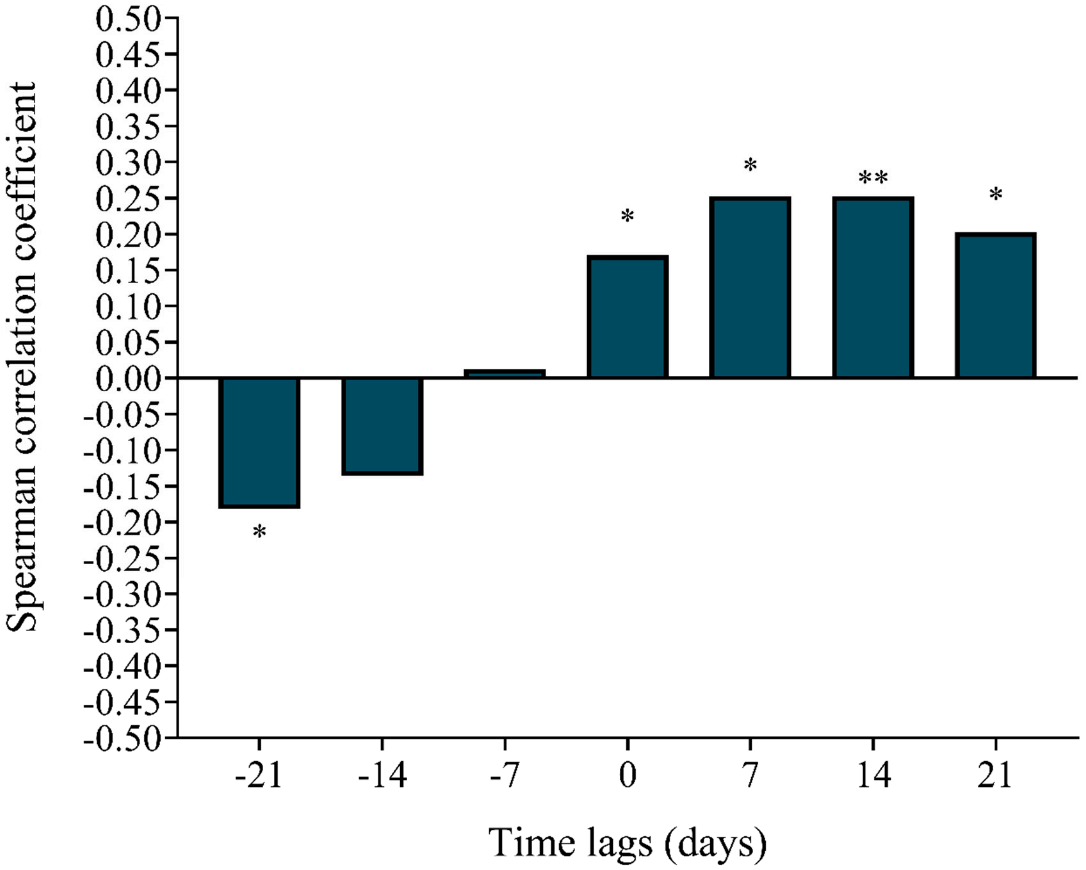

Figure S1. Time-lag correlations of global online search activity and daily new cases.
*P<0.05; **P<001.

Table S2. Time-lag correlations of online search activity and daily new cases in 102 countries or territories.

Countries or territories	Lag -21 days	Lag -14 days	Lag -7 days	Lag 0 days	Lag 7 days	Lag 14 days	Lag 21 days
World	-0.182*	-0.136	0.013	0.171*	0.253**	0.253**	0.202*
Andorra	0.085	-0.058	-0.057	0.071	-0.057	-0.060	0.066
Argentina	0.024	-0.029	-0.094	-0.094	0.011	-0.069	-0.083
Aruba	0.076	-0.067	-0.064	0.063	-0.064	0.047	0.062
Australia	0.065	0.036	0.013	0.123	0.095	0.086	0.058
Austria	-0.076	-0.037	-0.043	0.025	-0.001	-0.014	-0.064
Bahamas	0.030	0.027	-0.084	0.009	-0.083	0.005	-0.087
Bahrain	-0.056	0.167*	-0.054	0.157*	-0.054	-0.055	0.173*
Barbados	0.131	0.137	0.069	0.115	0.077	-0.053	-0.055
Belgium	-0.021	-0.035	0.124	0.195*	0.070	0.059	0.103
Benin	-0.037	-0.036	-0.035	-0.034	-0.034	-0.035	0.189*
Bermuda	0.189*	-0.044	-0.042	0.162*	0.190*	0.184*	-0.044
Bolivia	0.065	0.065	0.088	0.034	0.024	0.119	0.023
Bosnia and Herzegovina	-0.067	-0.064	-0.075	0.212**	-0.085	-0.085	0.001
Brazil	0.212*	0.213**	0.287***	0.350***	0.398***	0.426***	0.467***
Bulgaria	-0.115	-0.046	0.057	0.055	-0.038	-0.033	0.026
Cameroon	0.044	0.018	-0.093	0.005	-0.090	-0.093	-0.012
Canada	-0.088	-0.046	0.088	0.186*	0.206*	0.274***	0.220**
Chile	0.061	0.017	-0.025	0.053	-0.013	0.130	0.023
China	-0.132	-0.071	-0.007	-0.005	-0.053	-0.004	0.008
Colombia	0.014	0.075	0.005	0.152	0.215**	0.178*	0.114
Congo	-0.063	-0.063	-0.060	0.055	-0.058	-0.060	-0.049
Costa Rica	-0.077	0.035	0.161*	0.081	-0.010	-0.021	-0.023
Croatia	-0.074	0.046	-0.005	-0.034	-0.042	-0.060	0.027
Cuba	-0.049	-0.047	0.066	0.041	0.045	-0.068	0.060
Curacao	0.150	0.062	-0.071	-0.068	0.026	0.040	-0.077
Cyprus	0.081	-0.018	-0.056	0.042	0.051	0.059	0.050
Czechia	-0.013	-0.040	-0.165*	0.067	0.005	-0.009	-0.025
Democratic Republic of Congo	-0.015	-0.009	0.060	-0.016	-0.107	0.074	0.067
Denmark	-0.047	0.007	-0.017	0.057	-0.031	0.003	-0.147
Dominican Republic	-0.102	-0.041	-0.046	0.055	0.001	0.034	-0.015
Ecuador	-0.116	0.012	-0.078	-0.099	0.046	-0.048	0.038
El Salvador	0.197*	0.010	0.132	-0.111	0.138	-0.024	-0.021
Estonia	-0.061	-0.064	0.086	-0.078	0.044	-0.091	0.120
Finland	-0.102	-0.030	-0.049	0.024	-0.074	-0.026	0.020
France	-0.109	-0.036	-0.014	0.169*	0.125	0.044	0.024
Georgia	-0.067	-0.067	-0.065	0.201*	0.048	0.255**	0.071
Germany	-0.007	-0.026	-0.002	0.167*	0.087	0.043	0.011
Ghana	-0.115	0.021	-0.061	0.026	-0.082	-0.058	-0.054
Gibraltar	0.118	-0.078	-0.076	0.037	0.013	0.044	-0.079
Greece	0.117	0.024	-0.014	0.051	-0.118	-0.043	-0.085
Greenland	0.120	-0.042	-0.041	-0.040	-0.040	-0.042	0.159
Guadeloupe	-0.033	-0.034	-0.033	-0.032	-0.032	-0.032	-0.029

Guam	-0.052	-0.053	-0.050	0.173*	-0.048	-0.050	-0.051
Guatemala	0.073	0.025	0.084	0.132	0.200*	0.196*	0.098
Guyana	0.072	-0.073	-0.070	0.078	0.209**	-0.072	-0.073
Honduras	-0.024	-0.014	-0.046	0.062	-0.106	-0.038	0.120
Hungary	-0.185*	-0.084	-0.139	-0.032	-0.020	-0.069	0.146
Iceland	0.140	0.156	-0.011	-0.015	-0.021	0.021	0.203*
India	0.014	0.110	0.125	0.198*	0.130	-0.009	-0.017
Indonesia	-0.114	0.082	0.069	0.123	0.003	0.095	0.083
Iran	-0.066	-0.066	-0.062	-0.060	-0.062	0.087	-0.068
Ireland	-0.101	-0.092	0.056	0.087	0.120	-0.071	-0.051
Israel	0.030	0.078	-0.020	0.018	-0.037	-0.054	-0.067
Italy	-0.032	0.030	0.093	0.158*	0.095	0.047	0.042
Jamaica	-0.077	-0.165*	-0.095	0.101	-0.056	0.059	-0.015
Japan	-0.116	0.028	0.021	0.045	-0.041	0.052	-0.048
Jordan	-0.049	-0.049	0.116	-0.045	-0.048	-0.049	0.120
Latvia	0.029	-0.153	-0.146	0.090	0.087	0.033	0.074
Lebanon	-0.073	-0.128	0.006	-0.057	-0.012	-0.011	-0.093
Liberia	0.060	0.053	0.045	0.048	0.045	0.025	0.053
Lithuania	0.052	-0.100	-0.097	0.141	0.139	0.094	-0.104
Luxembourg	0.144	-0.073	0.085	-0.007	0.053	-0.032	0.110
Malta	-0.145	0.033	-0.027	-0.140	0.096	-0.087	-0.031
Martinique	-0.039	-0.040	-0.038	0.146	-0.040	0.133	-0.044
Mexico	0.157	0.038	0.023	-0.017	0.041	0.067	0.091
Moldova	-0.061	0.077	0.080	-0.058	0.249**	0.077	-0.061
Montenegro	-0.064	0.103	-0.060	0.244**	0.088	0.177*	-0.064
Morocco	-0.073	0.028	-0.072	0.212**	0.192*	0.185*	-0.080
Netherlands	0.038	0.000	0.025	0.191*	0.078	0.069	0.059
New Caledonia	-0.044	-0.042	-0.042	-0.040	-0.040	-0.040	-0.041
New Zealand	-0.026	0.082	-0.074	0.074	-0.021	-0.033	-0.027
Nigeria	-0.091	0.118	-0.033	0.012	0.083	-0.050	0.017
Norway	-0.095	-0.079	0.000	0.023	-0.001	-0.040	-0.022
Panama	-0.094	-0.066	-0.096	-0.019	-0.041	0.140	-0.036
Paraguay	0.163	0.131	-0.068	-0.064	-0.066	0.205*	0.200*
Peru	0.055	0.103	0.121	0.049	0.107	0.048	0.076
Philippines	0.100	0.088	0.129	0.181*	0.031	0.116	0.035
Poland	-0.106	0.002	-0.009	-0.037	-0.060	0.054	0.035
Portugal	-0.021	0.070	0.127	0.247**	0.175*	0.162*	0.097
Puerto Rico	-0.041	0.023	-0.035	0.070	0.013	0.077	0.130
Qatar	0.133	-0.013	0.021	0.131	0.105	-0.066	-0.151
Romania	-0.035	-0.005	-0.070	-0.043	0.132	0.015	-0.058
Russia	-0.085	-0.085	0.062	0.149	0.039	0.067	0.005
Saudi Arabia	-0.009	0.052	0.080	0.145	-0.001	0.112	0.156
Serbia	-0.031	0.016	0.023	0.014	0.062	-0.076	-0.032
Singapore	0.029	0.068	0.127	0.276***	0.097	0.074	0.081
Slovakia	0.010	0.009	-0.034	-0.086	0.065	-0.036	-0.068
Slovenia	-0.006	-0.077	-0.052	0.163*	0.171*	0.050	0.039
South Africa	-0.054	0.054	0.044	0.128	-0.043	0.081	0.146
South Korea	-0.090	0.037	-0.061	0.040	-0.030	0.059	0.024
Spain	-0.118	0.030	-0.010	0.144	0.044	0.128	0.101
Sudan	0.103	0.042	-0.029	0.048	-0.013	0.008	0.121
Sweden	-0.127	-0.054	-0.041	0.035	0.043	-0.024	-0.104

Switzerland	0.090	0.079	0.096	0.244**	0.198*	0.163*	0.213*
Thailand	-0.039	-0.005	0.029	0.051	0.069	0.061	0.022
Turkey	0.037	0.054	0.071	0.035	-0.046	-0.028	-0.060
Ukraine	-0.047	-0.002	-0.080	0.096	-0.093	0.005	-0.010
United Arab Emirates	0.043	0.081	0.219**	0.226**	0.144	0.027	-0.199*
United Kingdom	0.009	0.121	0.209**	0.286***	0.314***	0.149	0.016
United States	-0.071	0.001	0.095	0.194*	0.232**	0.275***	0.207*
Uruguay	0.097	-0.010	-0.001	0.033	0.043	0.038	-0.026
Venezuela	0.015	-0.090	-0.087	0.061	-0.098	0.013	-0.104

*P<0.05; **P<0.01; ***P<0.001
